# Effect of Continuous Intake of *Lactobacillus salivarius* WB21 on Tissues Surrounding Implants: A Double-Blind Randomized Clinical Trial

**DOI:** 10.3390/life14121532

**Published:** 2024-11-22

**Authors:** Yusuke Taniguchi, Nao Suzuki, Kae Kakura, Kazunari Tanabe, Ryutaro Ito, Tadahiro Kashiwamura, Akie Fujimoto, Marie Naito, Masahiro Yoneda, Takashi Hanioka, Hirofumi Kido

**Affiliations:** 1 Section of Oral Implantology, Department of Oral Rehabilitation, Fukuoka Dental College, 2-15-1 Tamura, Sawara-ku, Fukuoka 814-0193, Japan; yuusuke@fdcnet.ac.jp (Y.T.); okamatsu@fdcnet.ac.jp (K.K.); itor@fdcnet.ac.jp (R.I.); tadahiro@fdcnet.ac.jp (T.K.); hkido@fdcnet.ac.jp (H.K.); 2 Department of Preventive and Public Health Dentistry, Fukuoka Dental College, 2-15-1 Tamura, Sawara-ku, Fukuoka 814-0193, Japan; akief@fdcnet.ac.jp (A.F.); marie@fdcnet.ac.jp (M.N.); hanioka@tumh.ac.jp (T.H.); 3 Oral Medicine Research Center, Fukuoka Dental College, 2-15-1 Tamura, Sawara-ku, Fukuoka 814-0193, Japan; 4 Tanabe Preservative Dentistry, 2-12-18 Mizutani, Higashi-ku, Fukuoka 813-0031, Japan; tanabe-hozonshika@vivid.ocn.ne.jp; 5 Section of General Dentistry, Department of General Dentistry, Fukuoka Dental College, 2-15-1 Tamura, Sawara-ku, Fukuoka 814-0193, Japan; yoneda@fdcnet.ac.jp; 6 Faculty of Health Care Sciences, Takarazuka University of Medical and Health Care, 1 Hanayashiki-Midorigaoka, Takarazuka 666-0162, Japan

**Keywords:** *Lactobacillus salivarius*, periimplantitis, probiotics, randomized controlled trial

## Abstract

Objective: This study aimed to improve the health of peri-implant tissues through continuous intake of *Lactobacillus salivarius* WB21 (LSWB21) tablets. Methods: A double-blind, randomized controlled trial was conducted with 23 maintenance patients who had generally healthy oral peri-implant tissues. Participants were divided into a test group (*n* = 12) receiving LSWB21 tablets and a control group (*n* = 11) receiving placebos. All patients took one tablet three times daily for 2 months. Evaluation measures included modified Gingival Index (mGI), modified Plaque Index (mPI), modified Bleeding Index (mBI), salivary secretory IgA, and oral symptoms assessed at baseline, 1 month, and 2 months. Results: After 2 months, significant improvements in the mGI, mPI, and mBI were observed in the test group; significant improvement in the mPI was observed in the control group. Changes in the mGI over 2 months significantly differed between the groups (*p* = 0.038), and multiple regression analysis confirmed the effectiveness of LSWB21 in reducing the mGI (*p* = 0.034). Subjective symptoms such as bad breath in the test group and tongue symptoms in the control group also significantly improved. Conclusion: Continuous intake of LSWB21 may be beneficial for stabilizing peri-implant tissue. Trial registration: UMIN000039392 (UMIN-CTR).

## 1. Introduction

With an incidence exceeding 40%, peri-implantitis is the most frequent complication of oral implant treatment [1]. Because this inflammatory disease is associated with bacterial infection, typical periodontal therapies are used to eliminate inflammation. Non-surgical treatments, including mechanical plaque removal, local fungicide application, and systemic or topical antibiotic therapy, are used either alone or in combination, depending on the condition; however, patients rarely exhibit a complete cure without recurrence. Surgical treatment methods include excision therapy, gingival flap apical movement, implant-plasty, periodontal surgery, laser therapy, and regenerative therapy, but the evidence for these treatments remains insufficient. Furthermore, attempts are underway to establish usage protocols [2]. Considering the high incidence of peri-implantitis and the difficulty of healing, efforts to prevent its onset are needed.

Previous strategies to prevent peri-implantitis have been based on periodontitis prevention, which combines mechanical plaque control with chemical plaque control using mouthwashes and toothpaste that contain bactericidal and antibacterial ingredients. These preventive strategies address bacterial factors among the three types of epidemiological factors (bacterial factors, host factors, and environmental factors). Nevertheless, long-term use of mouthwashes and toothpaste containing bactericidal and antibacterial ingredients throughout the oral cavity cannot completely prevent the onset of peri-implantitis. The routine use of germicidal and antibacterial agents has become established in oral health management. However, reports indicate that the use of mouthwash containing antibacterial agents disrupts the balance of the oral microbiome and has systemic effects on the body, leading to issues such as increased blood pressure [3,4]. Consequently, new preventive strategies for oral care that do not entirely rely on antimicrobial approaches are currently in development.

In accordance with this trend, the use of lactic acid bacteria for oral health management is attracting attention [5]. Research on intestinal flora has revealed that lactic acid bacteria shift the resident flora to a healthy composition. The ingestion of lactic acid bacteria increases secretory IgA (SIgA) antibodies in the intestinal tract and saliva, and it is expected to prevent infectious diseases by improving immune function [6,7]. Clinical trials using lactic acid bacteria have been conducted for peri-implantitis [8,9,10,11,12]; some trials showed that the therapeutic effect was improved through the use of lactic acid bacteria [8,11], whereas others showed no difference from the control [9,10,12]. These differences among studies may have been influenced by the bacterial strain used, the frequency and duration of ingestion, the site of the implant studied, and the severity of peri-implantitis. Although lactic acid bacteria have various effects, those effects are mild; thus, if inflammation is already present, the effect of lactic acid bacteria may be difficult to observe when they are used in isolation.

*Lactobacillus salivarius* WB21 (LSWB21) reportedly has preventive effects on periodontal disease and bad breath, based on clinical experiments [7,13,14,15,16]. Because LSWB21 can potentially control inflammation and improve the oral microflora, it can enhance the health of peri-implant tissues. However, its effects on peri-implant tissues have not been investigated. This study, a double-blind, randomized, placebo-controlled trial, hypothesized that LSWB21 would effectively maintain the health of peri-implant tissues.

## 2. Methods

### 2.1. Study Population

A blinded, placebo-controlled, randomized controlled trial was conducted with 23 maintenance patients (9 men, 14 women, mean age 62.1 ± 12.3 years) who underwent implant treatment at the Department of Oral Implantology, Fukuoka Dental College Medical and Dental General Hospital and exhibited good peri-implant oral tissue conditions. All patients had keratinized tissue of 5 mm or more around the implant superstructure. The sample size was based on the minimum numbers reported in previous randomized controlled trials investigating the effects of probiotics on peri-implant tissues [6,8,10,11,12]. Exclusion criteria were edentulism, active treatment (excluding maintenance treatments), use of antibiotics within the previous 3 months, dairy allergy, use of other probiotics, severe metabolic disease (diabetes, renal disease, and liver disease), and malignant tumors. This research was conducted with the approval of the Fukuoka Gakuen Ethics Review Committee (Permit No. 498). The purpose, significance, method, and duration of the study were fully explained to the patients, and written consent to participate in the clinical trial was obtained from each patient with sufficient understanding.

### 2.2. Study Design

**Figure 1:**
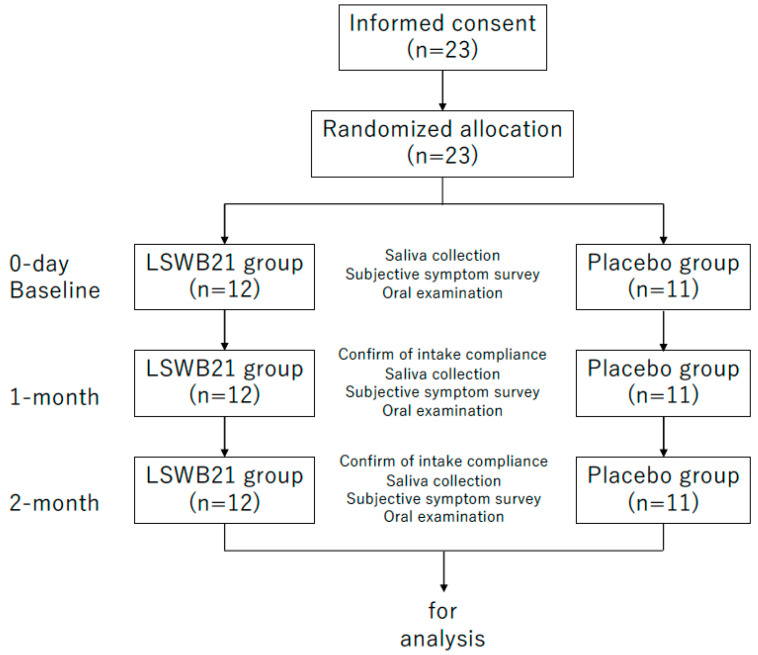
Flowchart of this randomized, double-blind, placebo-controlled comparative study.

LSWB21-containing tablets or placebo tablets were given to patients in an order determined by an allocation table created using the permuted block method. Random numbers were computer-generated by Professor M. Yoneda, Department of General Dentistry, Fukuoka Dental College. This study enrolled sequential participants who provided consent. The principal investigator, clinical examiner, and study staff responsible for patient contact and endpoint measurement were blinded to medication assignment until enrollment and data collection had been completed. The LSWB21 tablets (Wakamoto Pharmaceutical Co., Tokyo, Japan) used were circular with a diameter of 14 mm; the three tablets contained 2.0 × 10^9^ colony-forming units (CFU) of LSWB21, along with 840 mg of xylitol. Placebo tablets had the same taste, appearance, and texture, but the LSWB21 had been removed. Instructions for taking the tablets were to place one tablet on the tongue after each meal, three times per day, without chewing. The intervention period was 2 months. At baseline, 1 month, and 2 months, examinations of inflammation (modified Gingival Index, mGI), plaque (modified Plaque Index, mPI), and bleeding (modified Bleeding Index, mBI) at the implant site were conducted (Figure 1). All intraoral examinations were performed by a single dentist specializing in implantology. Resting saliva samples were collected before each oral examination to assess SIgA levels in saliva. Furthermore, patients were asked to self-evaluate their subjective symptoms over the previous week using a visual analog scale (VAS). During the intervention period, all patients used the same toothbrush (Ruscello^®^ I-20 Implant, GC, Tokyo, Japan) and dentifrice (Clinica LION Mild Mint; LION, Tokyo, Japan); they were not allowed to use toothpaste or mouthwash containing antibacterial ingredients.

### 2.3. Oral Examination

The measures mGI [17], mPI [18], and mBI [18] around the implant were scored as described in the literature; the mean value measured using a six-point method was considered the representative value for each individual. The mGI score range is 0–4 (0: absence of inflammation; 1: mild inflammation not affecting the entire marginal or papillary gingival unit; 2: mild inflammation of the entire marginal or papillary gingival unit; 3: moderate inflammation; 4: severe inflammation), and the mPI range is 0–3 (0: no plaque detected; 1: plaque only detected by running a probe across the smooth marginal surface of the implant; 2: plaque can be seen with the naked eye; 3: abundance of soft matter), and the mBI range is 0–3 (0: no bleeding when periodontal probe is passed along the gingival margin adjacent to the implant; 1: isolated spots of bleeding visible; 2: blood forms a confluent red line along margin; 3: heavy or profuse bleeding).

### 2.4. Measurement of Salivary SIgA

To assess SIgA levels in saliva, 1 mL of resting saliva was collected prior to each oral examination. A saliva collection kit (Saliva Collection Aid; Salimetrics, Carlsbad, CA, USA) was used for saliva collection. Saliva samples were stored at −20 °C until analysis. After all samples had been collected, salivary SIgA was quantified by enzyme immunoassay (EIA) using the Salivary Secretory IgA EIA Kit (Salimetrics).

### 2.5. Subjective Symptoms Related to the Oral Cavity Assessed Using a VAS

At each examination (baseline, 1 month, and 2 months), patients were asked to assess their subjective oral symptoms over the previous week using a VAS. The questions included (1) sticky feeling in the mouth, (2) bleeding with brushing, (3) feeling that teeth are floating, (4) itchy or painful gums, (5) bad breath, (6) the surface of the tongue is white, (7) slimy feeling on the tooth surface, and (8) feeling tooth pain with cold water.

### 2.6. Statistical Analysis

For comparisons between the two groups at baseline, the Pearson chi-square test was used for the male–female ratio and implant site; the Mann–Whitney *U* test was used for all other comparisons. The normality of the data distribution was evaluated using the Shapiro-Wilk test. The Friedman test was used to analyze changes in outcomes among the baseline, 1-month, and 2-month screenings in each group. Changes from baseline to 1 month and from baseline to 2 months were evaluated using the Wilcoxon signed rank test; their statistical significance was determined using the Bonferroni correction method. The extent of changes in outcomes between the LSWB21 and placebo groups over the 2-month period was analyzed using the Mann–Whitney *U* test. In multiple regression analysis with changes in the mGI as the dependent variable, age, sex, and LSWB21 consumption (yes/no) were utilized as independent variables. Furthermore, considering the significant difference in the mGI between the LSWB21 and placebo groups at baseline, multiple regression analysis was performed using the weighted least squares (WLS) method, which is used to overcome the challenges associated with heteroscedasticity [19]. Changes in subjective oral symptoms in each group during the 2-month period were analyzed using the Friedman test. Within-group differences from baseline to 1 month and from baseline to 2 months were evaluated using the Wilcoxon signed rank test; their statistical significance was determined using the Bonferroni correction method. SPSS software (Version 27.0; SPSS Japan, Tokyo, Japan) was used for statistical analyses. In all analyses, *p* < 0.05 was regarded as the threshold for statistical significance.

## 3. Results

### 3.1. Characteristics of the Study Population

Table 1 lists the characteristics of the study participants. At baseline, participants’ peri-implant mGI score was lower than 2 (corresponding to mild inflammation) in both groups, but it was significantly higher in the LSWB21 group (median score 1.67) than in the placebo group (median score 1.17) (*p* = 0.023). The median mPI score was less than 1 in both groups, the mBI score was nearly 0, and peri-implant conditions were generally good in all patients. Compliance with tablet intake was 89.1% in the LSWB21 group and 86.7% in the placebo group; it did not significantly differ between the two groups.

### 3.2. Outcome Analysis

Table 2 lists the baseline, 1-month, and 2-month outcome values for the LSWB21 and placebo groups. In the LSWB21 group, mGI, mPI, and mBI values significantly decreased over the 2-month observation period; they also significantly decreased in the first month. In the placebo group, the mPI significantly decreased over the 2-month period. A significant difference between the LSWB21 and placebo groups was observed in the extent of changes in the mGI over 2 months (*p* = 0.038). Salivary SIgA slowly increased in both groups over the 2-month study period, but no statistically significant difference was observed.

Table 3 lists the results of multiple regression analysis of changes in the mGI. The presence or absence of LSWB21 intake had a significant effect on changes in the mGI (*p* = 0.014). Next, considering the significant difference in mGI scores between the LSWB21 and placebo groups at baseline, changes in the mGI analyzed using the weighted least squares method were significantly influenced by the presence or absence of LSWB21 intake (*p* = 0.034).

### 3.3. Changes in Subjective Symptoms in the Oral Cavity

Table 4 presents the results regarding subjective symptoms in the oral cavity. In the LSWB21 group, awareness of “bad breath” significantly decreased (*p* = 0.012). Significant differences were observed between baseline and 1 month, as well as between baseline and 2 months. In the placebo group, significant decreases in “the surface of the tongue are white” (*p* = 0.007) and “slimy feeling on the tongue surface” (*p* = 0.010) were recorded. A significant difference was found in “the surface of the tongue is white” between baseline and 1 month.

## 4. Discussion

Because no treatment is currently established for peri-implantitis, preventive management of peri-implant mucositis is considered the most important approach for preventing the onset of peri-implantitis [20]. As bacterial plaque is the etiological agent of peri-implantitis, effective plaque control, regular professional mechanical debridement, smoking cessation, and maintaining optimal blood glucose levels are essential preventive measures [21]. Considering that probiotics balance oral microflora and influence host immunity, incorporating probiotics alongside these preventive measures may support peri-implant tissue health. Accordingly, the present study aimed to use lactic acid bacteria to maintain and improve the health of peri-implant tissues, targeting patients with peri-implant tissue statuses that ranged from clinically stable health to mild peri-implant mucositis. The results show that continuous intake of LSWB21 may be effective for maintaining the health of peri-implant tissues. In the LSWB21 group, the mGI, mPI, and mBI all showed significant improvements in the first month of the 2-month intervention period; they subsequently showed gradual improvement or maintained low values. Conversely, in the placebo group, despite significant decreases in numerical values of the mPI over the 2-month observation period, no statistically significant changes were observed in the first month of the 2-month intervention period. Regarding the effects of LSWB21 on oral health, improvements in periodontal pockets and bleeding on probing were confirmed in a randomized controlled trial involving a 2-week intervention for patients with periodontal disease and oral malodor [14,16]. However, when bleeding on probing and plaque adhesion was evaluated in a 2-month intervention crossover study of healthy older people [7], a non-significant reduction was observed with LSWB21 intake. Although that study [7] and the present study both involved 2-month interventions in healthy individuals without oral inflammatory disease, the effect of LSWB21 intake was more pronounced in the present study. The major difference is that the previous study examined the periodontal tissues of natural teeth, whereas the present study examined tissues surrounding implants. Healthy peri-implant biofilms show minor differences in taxonomic composition compared with adjacent healthy natural teeth, but they have a less diverse microbiome [22]. Another difference is that whereas the previous study [7] evaluated the entire oral cavity by examining several representative teeth, this study meticulously examined the area around a single implant.

Salivary SIgA levels gradually increased during the study period, but no difference was observed between the LSWB21 and placebo groups. Many clinical studies targeting older people have shown that ingestion of lactic acid bacteria increases salivary SIgA levels [7,23,24,25]. In contrast, studies targeting younger people have shown no effect [26]. Our patients were in the range of early older age and generally were healthy. Furthermore, in contrast to previous research [24], we did not select participants based on salivary SIgA levels. Salivary SIgA exhibits circadian changes and is easily influenced by stress and exercise [27,28]. Although the present study was designed to test each patient at the same time of day, test appointment times varied among patients; thus, not all patients were tested at the same time.

Few major differences in subjective oral symptoms were observed between the LSWB21 and placebo groups (Table 4). This lack of response may have been due to the slow rate of action of lactic acid bacteria and the fact that this study targeted patients with stable oral environments. To ensure that patients voluntarily continue using lactic acid bacteria, it is important that the effects are clearly observable. A significant improvement in the subjective symptom of bad breath was observed in the LSWB21 group. Regarding the halitosis-suppressing effect of LSWB21, a crossover study of patients with halitosis showed decreases in organoleptic test scores and the concentrations of volatile sulfur compounds, which are the main substances responsible for oral malodor [16]. Halitosis is a symptom that many people are concerned about, and it has been reported that feedback from halitosis measurements is effective in helping male smokers quit smoking [29]. Continued use of LSWB21 may be promoted through health guidance that mentions the halitosis-suppressing effect of LSWB21.

The purpose of this study was to verify the preventive effect of LSWB21 on peri-implantitis; however, none of the patients in either group (LSWB21 or placebo) developed peri-implantitis during the 2-month intervention period. To elucidate the preventive effect of LSWB21 on peri-implantitis, continued prognostic monitoring of the patients is needed. However, greater improvements in the mGI, mPI, and mBI were observed in the LSWB21 group compared to the placebo group, indicating that supplementary use of lactic acid bacteria as self-care could maintain the health of tissues surrounding the implant. In the future, initiating probiotic therapy before implant treatment may improve the success of implant treatment and its prognosis.

A limitation of this study was the small sample size. Although the number of participants was comparable to those of previous studies investigating the effects of probiotic intake on peri-implant tissues, participant allocation was determined based on the order of consent to participate. This led to a baseline difference in mGI between the experimental and placebo groups. Therefore, although the WLS method was employed in the multiple regression analysis, the allocation should have ensured similar baseline conditions across groups.

## 5. Conclusions

To evaluate the effect of LSWB21 on maintaining the health of tissues around oral implants, we conducted a double-blinded, placebo-controlled, randomized clinical trial with 23 maintenance patients who exhibited relatively good tissue conditions around their oral implants. In the LSWB21 group (*n* = 12), the mGI, mPI, and mBI improved over 2 months; in the placebo group (*n* = 11), the mPI improved during the same period. A statistically significant difference concerning the changes in mGI over 2 months was observed between the two groups. These results imply that continuous intake of LSWB21 can stabilize peri-implant tissues.

## Figures and Tables

**Table 1:** Characteristics of the study population (number or median [IQR]).

	LSWB21 (*n* = 12)	Placebo (*n* = 11)	*p*-Value
Age (years)	63.0 (59.3, 73.0)	60.0 (51.2, 71.0)	0.288
Female/Male (*n*)	8/4	6/5	0.552
Number of brushing (/day)	2.0 (2.0, 2.6)	3.0 (1.8, 3.0)	0.740
Clinical findings			
mGI	1.67 (1.38, 1.83)	1.17 (1.00, 1.50)	0.023
mPI	0.83 (0.50, 1.29)	0.50 (0.33, 1.17)	0.288
mBI	0.17 (0.00, 0.63)	0.17 (0.00, 0.33)	0.487
Implant site			
Anterior teeth (*n*)	1	0	0.391
Premolars (*n*)	4	2	
Molars (*n*)	7	9	
Compliance (%)	89.1 (84.4, 92.3)	86.7 (83.4, 87.8)	0.288

Female/male and implant sites were analyzed using the Pearson chi-square test. The remaining items were analyzed using the Mann–Whitney *U* test.

**Table 2:** Baseline, 1-month, and 2-month outcome values for the LSWB21 (*n* = 12) and placebo (*n* = 11) groups (median [IQR]).

Items	Group	Baseline	1 Month	2 Months	*p-*Value ^a^	LSWB21 vs. Placebo ^b^
mGI	LSWB21	1.67 (1.38, 1.83) ^c,d^	1.08 (0.75, 1.63) ^c^	0.50 (0.21, 0.96) ^d^	0.000	0.038
	Placebo	1.17 (1.00, 1.50)	1.17 (0.67, 1.33)	0.67 (0.50, 1.00)	0.058	
mPI	LSWB21	0.83 (0.50, 1.29) ^c,d^	0.33 (0.17, 0.33) ^c^	0.25 (0.17, 0.50) ^d^	0.001	0.200
	Placebo	0.50 (0.33, 1.17)	0.33 (0.33, 0.50)	0.33 (0.00, 0.83)	0.021	
mBI	LSWB21	0.17 (0.00, 0.63) ^c^	0.00 (0.00, 0.00) ^c^	0.00 (0.00, 0.00)	0.001	0.171
	Placebo	0.17 (0.00, 0.33)	0.00 (0.00, 0.00)	0.00 (0.00, 0.00)	0.062	
SIgA (log µg/mL)	LSWB21	2.22 (2.07, 2.48)	2.30 (1.95, 2.41)	2.48 (2.08, 2.66)	0.368	0.204
	Placebo	2.28 (2.14, 2.53)	2.30 (2.12, 2.60)	2.34 (2.11, 2.48)	0.913	

^a^ Repeated measures analysis of variance within a group was performed using the Friedman test. ^b^ Mann–Whitney *U* test was used to compare the change in outcomes over 2 months between the two groups. ^c^ *p* < 0.0167 (Bonferroni correction) between baseline and 1 month using the Wilcoxon signed rank test. ^d^ *p* < 0.0167 (Bonferroni correction) between baseline and 2 months using the Wilcoxon signed-rank test.

**Table 3:** Multiple regression analysis of mGI changes over 2 months.

	Model 1				Model 2			
Predictor	B	95% Confidence Interval	β	*p-*Value	B	95% Confidence Interval	β	*p-*Value
Age	0.011	−0.007, 0.028	0.262	0.212	0.010	−0.008, 0.027	0.243	0.270
Sex	0.515	−0.077, 0.763	0.343	0.103	0.297	−0.120, 0.714	0.307	0.153
Group	0.517	0.115, 0.919	0.530	0.014	0.464	−0.039, 0.889	0.483	0.034

Model 2 used the weighted least squares method.

**Table 4:** Changes in subjective oral symptoms (range 0–100, average ± standard deviation).

	LSWB21 Group (*n* = 12)				Placebo Group (*n* = 11)			
Item	Baseline	1 Month	2 Months	*p* Value ^a^	Baseline	1 Month	2 Months	*p* Value ^a^
Sticky feeling in the mouth	16.5 ± 14.4	8.9 ± 11.7	13.7 ± 19.1	0.452	21.7 ± 22.8	10.7 ± 10.2	13.4 ± 20.3	0.105
Bleeding during brushing	7.4 ± 18.5	6.2 ± 15.6	7.7 ± 14.4	0.347	13.2 ± 21.4	5.6 ± 13.1	5.0 ± 7.5	0.394
Feeling that teeth are floating	8.1 ± 12.1	4.8 ± 8.2	8.4 ± 13.1	0.192	3.4 ± 3.6	4.1 ± 7.4	3.3 ± 3.5	0.764
Itchy or painful gums	7.5 ± 10.3	6.2 ± 9.5	7.6 ± 8.1	0.614	4.3 ± 3.8	2.7 ± 5.1	4.8 ± 5.8	0.733
Bad breath	30.1 ± 24.3 ^b,c^	15.6 ± 23.1 ^b^	14.1 ± 17.2 ^c^	0.012	24.3 ± 30.5	17.8 ± 19.2	9.5 ± 14.3	0.061
Tongue surface is white	31.7 ± 33.9	19.2 ± 26.7	20.7 ± 23.3	0.352	36.2 ± 36.0 ^b^	17.0 ± 22.4 ^b^	19.6 ± 24.8	0.007
Slimy feeling on the tooth surface	15.4 ± 17.0	9.5 ± 10.9	9.4 ± 10.3	0.846	24.8 ± 27.3	11.3 ± 15.6	9.4 ± 15.8	0.010
Toothache caused by cold water	21.3 ± 26.8	10.0 ± 11.3	10.6 ± 12.6	0.898	17.3 ± 26.3	10.4 ± 17.0	7.4 ± 12.7	0.435

^a^ Repeated measures analysis of variance within a group was performed using the Friedman test. ^b^ *p* < 0.0167 (Bonferroni correction) between baseline and 1 month. ^c^ *p* < 0.0167 (Bonferroni correction) between baseline and 2 months.

## Data Availability

The data presented in this study are available upon request from the corresponding author. The data are not publicly available due to restrictions, e.g., privacy or ethical concerns.
